# Supplementary material for: Design and Synthesis of Bio-Inspired Polyurethane Films with High Performance
Source: Polymers (Basel). 2020 Nov 17;12(11):2727. doi: 10.3390/polym12112727 (PMC7698539; doi:10.3390/polym12112727)
Supplement: Supplementary file 1 [file polymers-12-02727-s001.zip › polymers-984348-supplementary.docx]

Electronic supplementary information for:

Design and synthesis of bio-inspired polyurethane coatings with high performance

Eva Marina Briz-López,^a^ Rodrigo Navarro,^*b,c^ Héctor Martínez-Hernández,^a^ Lucía Téllez-Jurado^a^ and Ángel Marcos-Fernández^b,c^

a. Instituto Politécnico Nacional-ESIQIE, Dpto. Ing. En Metalurgia y Materiales, UPALM-Zacatenco, Mexico City, 07738, Mexico.

b. Institute of Polymer Science and Technology (ICTP-CSIC), Juan de la Cierva, 3. 28006, Madrid, Spain.

c. Interdisciplinary Platform for “Sustainable Plastics towards a Circular Economy” (SUSPLAST-CSIC), Madrid, Spain.

Figure S1: A) ATR-FTIR spectrum between 4000 to 450 cm^-1^ and B) 13C-NMR spectrum of functionalized catechol.

Figure S2: DSC thermogram of pristine PCL-2000 with molecular weight of 2037 g/mol.


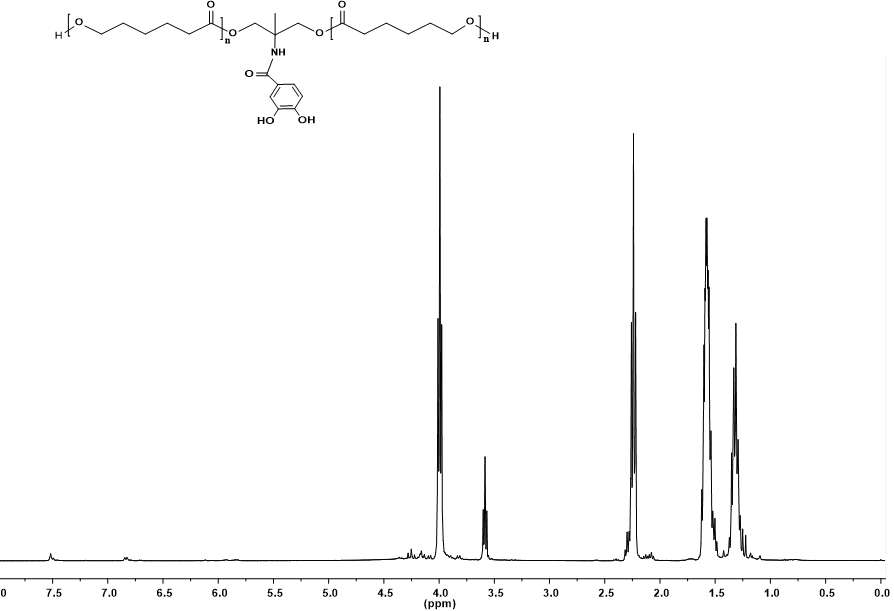


Figure S3: ^1^H-NMR spectrum of Poly(ε-caprolactone) bearing catecholic moieties (PCL-Cat).

Figure S4: a) ^1^H-NMR spectrum of functionalized segmented polyurethane (PU-Cat-SS 20) with catechol units within soft-segment. B) Comparison between ATR-FTIR spectra of PU-Cat-HS 20 and PU-Cat-SS 20.

Figure S5: ^1^H-NMR (A) and ^13^C-NMR (B) spectra of orthoformate protected Protocatechuic acid.

Figure S6: ^1^H-NMR (A) and ^13^C-NMR (B) spectra of protected catechol-diol in deuterated DMSO.


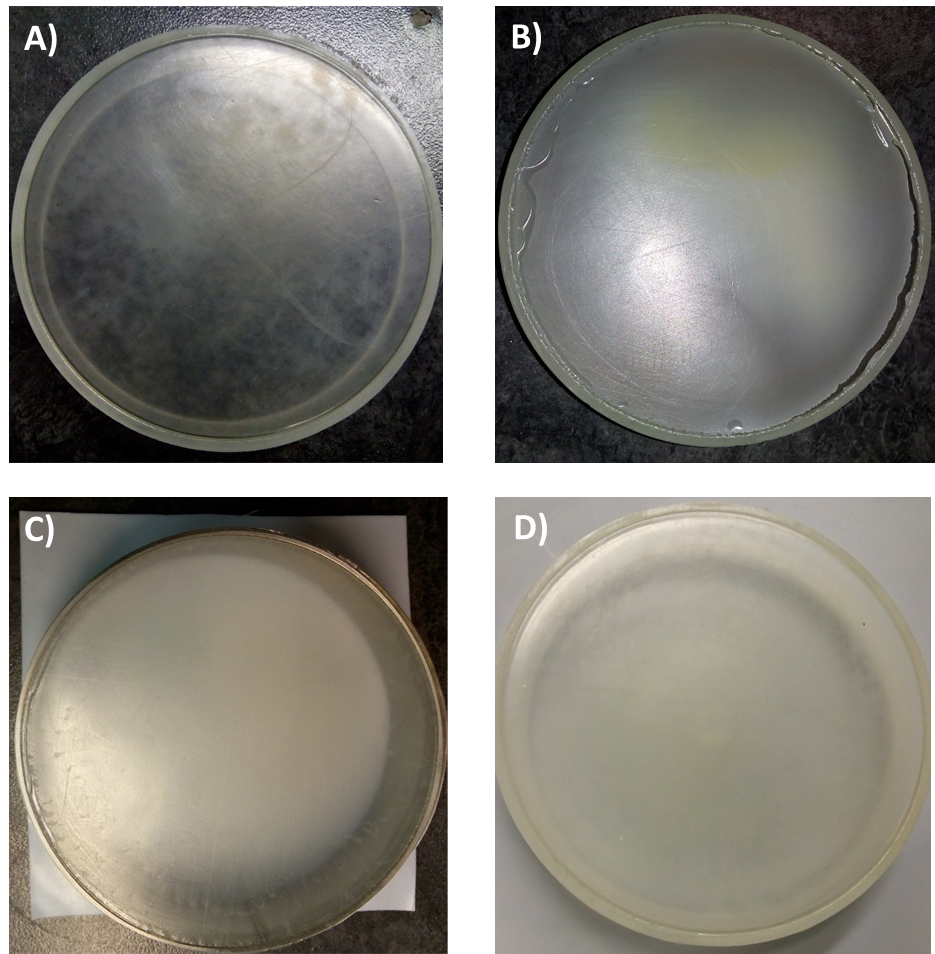


Figure S7: Pictures of studied polyurethanes. A) Pristine PU, B) PU-Cat-HS 20, C) PU-Cat-SS 20, D) PU-Cat_prot_-HS 20

**Table S1:** Thermal degradation of polyurethanes with **Cat-Fun** as chain extender

| **Sample** | **T_d1_ (ºC)** | **Weight loss (%)** | **T_d2_ (ºC)** | **Weight loss (%)** | **Residue  (%)** |
| --- | --- | --- | --- | --- | --- |
| **PU-Cat-HS 20** | 230 | 5.4 | 374 | 92.8 | 1.8 |
| **PU-Cat-HS 30** | 207 | 11.5 | 360 | 87.0 | 1.5 |
| **PU-Cat-HS 40** | 210 | 22.1 | 368 | 76.2 | 1.7 |
